# Ensuring Nutritional Security in India through Wheat Biofortification: A Review

**DOI:** 10.3390/genes13122298

**Published:** 2022-12-06

**Authors:** Umesh Kamble, Chandra Nath Mishra, Velu Govindan, Amit Kumar Sharma, Sushma Pawar, Satish Kumar, Gopalareddy Krishnappa, Om Prakash Gupta, Gyanendra Pratap Singh, Gyanendra Singh

**Affiliations:** 1 ICAR-Indian Institute of Wheat and Barley Research, Karnal 132001, India; 2 CIMMYT, Texcoco 56237, Mexico; 3 ICAR-Sugarcane Breeding Institute Coimbatore, Veerakeralam 641007, India; 4 ICAR-National Bureau of Plant Genetic Resources New Delhi, New Delhi 110012, India

**Keywords:** food security, seed system, biofortification, meta-quantitative trait loci

## Abstract

Undernourishment of nutrients, also known as hidden hunger, affects over 2 billion populace globally. Even though stunting among children below five years of age has decreased in India in the last ten years, India is home to roughly thirty percent of the world’s population of stunted pre-schoolers. A significant improvement has been witnessed in the targeted development and deployment of biofortified crops; approximately 20 million farm households from developing counties benefit from cultivating and consuming biofortified crops. There is ample scope for including biofortified varieties in the seed chain, ensuring nutritional security. Wheat is a dietary staple in India, typically consumed as wholemeal flour in the form of flatbreads such as *chapatti* and *roti*. Wheat contributes to nearly one fifth of global energy requirements and can also provide better amounts of iron (Fe) and zinc (Zn). As a result, biofortified wheat can serve as a medium for delivery of essential micronutrients such as Fe and Zn to end users. This review discusses wheat biofortification components such as Fe and Zn dynamics, its uptake and movement in plants, the genetics of their buildup, and the inclusion of biofortified wheat varieties in the seed multiplication chain concerning India.

## 1. Introduction

Providing healthy and nutritionally rich food in sufficient quantities is a major United Nations Sustainable Development Goal (SDG) to end hunger by 2030. The imperative part of food security is to assure the nutritional security of the population. Only food-based approaches effectively overcome the adverse effects of malnutrition [1]. As per the SDG Report (2020), eliminating undernourishment remains a major challenge and the food insecurity situation has been aggravated by the COVID-19 crisis worldwide [2]. Micronutrient deficiencies affect 2 billion people worldwide [3]. Vitamin A, Fe, and Zn deficiencies are linked to more than half of all under-five-year-old deaths worldwide [4]. Micronutrient deficiencies are a type of malnutrition that occur when intake and accumulation of essential vitamins and minerals such as Fe, Zn, and iodine are too low to support overall health and well-being [5]. In 2019, the Food and Agriculture Organization (FAO) reported that over 200 million people in India were malnourished [6]. Therefore, various approaches, viz., diversifying food intake, supplying medical aid, and fortifying foods, are currently deployed to fight against malnutrition. These approaches, however, have several limitations, such as the lack of nutrient-enriched food for the poor sections of society, particularly the rural population. Biofortified crops continue to be one of the mostsuitable options for supplying the desired magnitude of micronutrients in a natural, durable, and economic manner [7,8,9,10,11].

**Figure 1:**
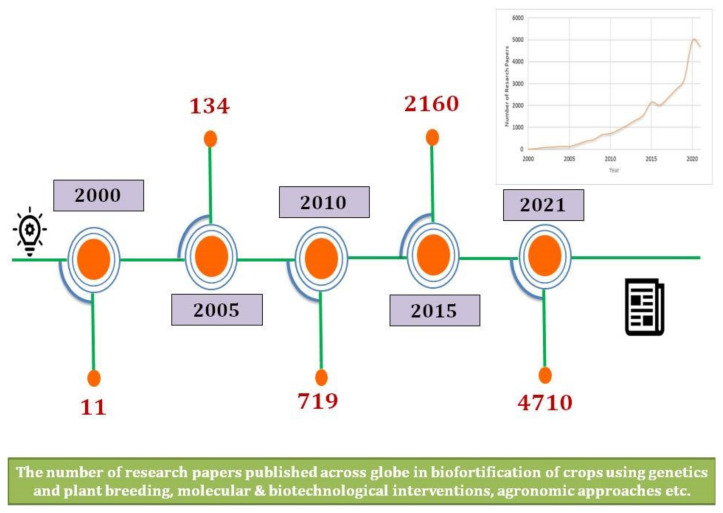
Escalating research on biofortification in the last two decades.

Biofortification is a sustainable strategy and harmonizing solution to malnutrition that involves breeding food crops for enhanced micronutrients with promising yields [12]. Research surroundingthe nutrition of food crops has become one of the primary research focuses in the last decade, as evidenced by the growing number of published articles on “biofortification” (Figure 1).

A high amount of genetic variability is present in the food for essential nutrients and for the development of high-yielding biofortified varieties using traditional or modern plant breeding approaches [13]. Researchers have worked to improve the Zn and Fe concentration of indigenous wheat varieties, resulting in the release of several biofortified varieties for commercial cultivation over the last five years, particularly in South Asia. As biofortified varieties are introduced into the seed and food systems, enhanced nutrition is naturally available in subsequent years without additional cost to farmers and consumers [11,14]. The genetic gain achieved by breeding programs is considered successful only when reasonably priced seeds of newly released varieties are provided to growers in a stipulated time. Breeder seed is the basic seed in the seed multiplication chain, is responsible for effective multiplication into certified seed, and is supplied to farmers for commercial cultivation [15]. India’s seed production system follows a well-defined protocol that considers countrywide and regional estimations of seed requirement [16]. National breeder seed indents depict the level of inclusion of newly released varieties in the seed multiplication chain. Thus, they can be employed as an indicator for assessing the promotion of biofortified varieties at the apex point of the seed chain. Therefore, a robust seed system for promoting biofortified varieties is essential in addressing micronutrient malnutrition.

## 2. Nutritional Security Status of India

With India being the second most densely inhabited nation on the planet, assuring food security to an ever-growing population is a vital challenge faced by agricultural scientists and policymakers. Although India has achieved self-sufficiency in total food grain production, food availability and nutritional status show regional disparities. In India, half of females of reproductive age are anemic and one third of children below five years are stunted [17]. Furthermore, according to the consumption data report, diets in India differ considerably from the EAT-Lancet reference diet across states and income groups [18]. The EAT-Lancet Commission recommends 2500 kcal/capita/day for all groups, with whole grains providing 811 kcal/capita/day [19]. India primarily produces wheat and rice as staple food crops for direct consumption. Whole grains share around 47% of the total calories consumed by the usual Indian household, with cereals accounting for up to 70% of the total calories consumed by the rural segment [18]. 

However, as per a World Health Organization (WHO) report, the disease burden of iron-deficiency anemia (IDA) in India was 53.4% in children (0–5 years) and 53.0% in women (15–49 years) [20], indicating opportunities for strategic interventions such as biofortification to combat micronutrient malnutrition. According to a national nutrition survey conducted between 2016 and 2018, approximately 18.9% of children aged 1 to 4, 16.8% of children aged 5 to 9, and 31.7% of children aged 10 to 19 are Zn deficient [21]. Although various approaches such as food fortification, medical supplementation, dietary diversification and crop biofortification are recommended to tackle micronutrient malnutrition, genetic improvement of nutrients in crops is considered a sustainable and economic strategy as nutrients are available in natural form and there is no need for infrastructural change or an elaborate distribution system.

## 3. Biofortification of Wheat

Wheat (*Triticum aestivum* L.) is an important food crop that accounts for 17% of calorie intake and 20% of protein intake for over 2 billion people worldwide [22]. Wheat breeding programs have traditionally focused on increasing yield gains. After achieving sufficiency in food grain production, breeding for improved quality has become a significant research focus. Breeding for quality, previously based solely on phenotyping, has evolved into a more detailed genetic approach [23]. The potential of food to offer enough recommended nutrients for human health development is referred to as nutritional quality. Although wheat provides various micronutrients and other bioactive components, this is not enough to meet the daily requirement where wheat is the primary source of energy. The genetic variability for micronutrient availability in gene pools needs to be utilized following traditional and modern plant breeding approaches to develop nutri-rich varieties that are more cost-effective and have higher consumer preferences. Transgenic approaches could also be viable because rapid detection and description of genes involving high micronutrient content are required to engineer plant metabolism and breed nutri-rich wheat varieties. However, transgenic methods face significant hurdles, including complex regulatory issues and low consumer acceptance in some regions of the world [24]. The success of conventional plant breeding depends on the utilization of available genetic variability and the selection of advantageous plants in F_1_ and subsequent segregating generations. Plant breeders must look for alternative methods to improve crop nutritional profiles if there is insufficient genetic variation of desired micronutrient content in germplasm.

Breeding for micronutrient-dense wheat is a novel approach to micronutrient delivery, while farmer acceptance of biofortified cultivars and the deployment of new biofortified varieties into the wheat growing areas will be critical in combating micronutrient malnutrition [25]. Table 1 shows the baseline level of micronutrients compared to traditional cultivars and the higher levels of nutrients achieved in wheat. Globally, the CIMMYT (International Maize and Wheat Improvement Center) is leading a collaborative program under the HarvestPlus project to develop wheat varieties with 40% moreZn concentrations than cultivated varieties in the target environments of South Asia [26]. To date, 422 crop varieties within 12 target crops have been notified for commercial cultivation in 41 countries, with 22 varieties released as Zn-rich wheat under the HarvestPlus project [27].

### 3.1. Acquisition in Plants

Zn acquisition in plants is influenced significantly by edaphic factors such as soil alkalinity (pH), phosphorus (P) fertilization, other organic matter, cations, and plant factors, e.g., fungal association. Zn availability is influenced by soil alkalinity because of the development of insoluble complexes. Zn (0.5 mg kg-1 DTPA-extractable) is deficient in alkaline calcareous soils. The construction of hydrolyzed Zn and precipitation along with iron oxide (FeO) and adsorption on soil colloids are all caused by elevated soil pH [28]. When the pH of a soil solution increases from 5.5 to 7, the Zn concentration in the solution decreases by 45 times [29,30]. Soils with a pH more than 8 and with an FeO coating around carbonate minerals have better sorption capability than discrete phases, further complicating Zn availability [31]. Iron oxides significantly affect heavy metal balances in soil solutions due to their presence as coatings and concretions, as well as discrete colloidal particles with a strong affinity for metal ions [32]. The coating formation of Fe is facilitated by the presence of carbonate minerals [33]. High P levels are a supplementary stress reason that could increase nutrient imbalances in cereal crops [34]. P fertilization also affects plant micronutrient availability; a higher dose of phosphorus decreases Zn content in grains by 17% to 56%, whereas other micronutrients such as Fe, copper (Cu), and manganese (Mn) are generally stable or even increased in the grain [35]. Excess P in Zn-deficient soils aggravates Zn deficiency and crop development [36,37]. Lime content typically impedes P availability, whereas Zn availability is largely determined by soil alkalinity.

The effect of low Zn levels in wheat was studied, and the research revealed that increasing P levels caused a significant reduction in Zn content in economic parts [34]. However, the antagonistic effect of phosphorus was moderated using mycorrhizae association, which enhanced the Zn assimilation via itswell-developed rooting system [36]. Arbuscular mycorrhizae (AM) enhanced plants’ Zn uptake by extending the plants’ root surface area and extensive soil volume [38,39]. Plants with AM associations showed elevated Zn concentrations [40]; specifically, plants cultivated on problematic soils utilized this symbiotic pathway to slowly accumulate plant nutrients such as phosphate, copper (Cu^2+^), and Zn^2+^ [41]. Symbiosis improves root morphology, makes nutrient uptake easier, and allows the host plant to withstand seasonal stress [42]. Mycorrhizae proliferation is reduced by P application, which reduces Zn accumulation in crops even further [43]. It has been reported in alkaline calcareous soils that the application of a higher dose of phosphorus (100 kg/ha) reduced zinc accumulation extensively in wheat. In contrast, Zn uptake was enhanced appreciably with mycorrhizae symbiosis [44].

### 3.2. Iron-Zinc Dynamics: Uptake and Translocation

**Figure 2:**
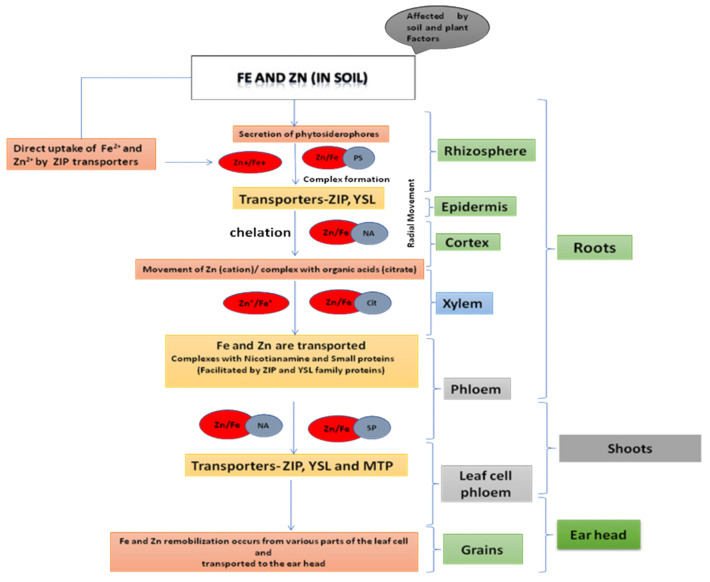
Molecular mechanism involved in the iron and zinc movement from soil to seed in the cereals.

Much research has been conducted to decipher the different paths of Fe and Zn from soil to grain in crops such as rice, maize, and barley. The homology of these inter-specific routes helps in understanding the Fe and Zn transport system in wheat, which is less understood [45]. The mechanism of nutritional uptake differs not only for plant species [46] but there are also differences among wheat genotypes for Zn and Fe absorption and further movement in the plant. There are two processes for Fe and Zn uptake: (i) Fe2+ and Zn2+ by ZIP (zinc–iron Permease family/ZRT-, IRT-like proteins) transporters are directly taken up and (ii) through the release of phytosiderophores (PSs) that bind Fe and Zn cations and are then transported by yellow stripe-like (YSL) [47]. The second strategy is followed in monocots such as wheat for Fe uptake (Figure 2). Fe and Zn uptake and transportation is carried out through many steps involving transporters from the same protein family; however, plants treat the two metals separately, often involving different elements of multi-gene families. Nicotianamine (NA), a metal chelator, plays an essential function in the radial movement of Fe and Zn through the root [48,49] and Zn transportation in t vacuoles influences by and large Zn transport from roots to shoots [50,51]. The transportation of Fe and Zn occurs through the xylem, where Zn flows as a cation or forming complex with organic acids (citrate) [52], and iron is generally chelated by the citrate [48]. The movement to phloem from xylem takes place in the root or basal shoot portion, or at the time of grain filling in leaves, and the process is smoothened by the proteins of the ZIP and YSL families. Because the xylem is discontinuous, all nutrients in wheat enter the grain via the phloem [53]. Fe and Zn are transported in the phloem as complexes with nicotianamine (NA) or smaller proteins. The aleurone layer, removed during milling, contains the major portion of Fe and Zn in wheat grain. The Fe from these tissues has less availability as deposition is mainly phytate-bound protein storage vacuoles (PSVs) [54]. Ferritin is thought to be more bioavailable and found in endosperm amyloplasts, which are widely consumed [55]. As a result, tissue localization and speciation are equally important along with total grain Fe and Zn content that affect the bioavailability.

### 3.3. Bioavailability

The fraction of a consumed micronutrient that is available for basic physiological functions or storage is referred to as bioavailability [56]. However, biofortification and food supplementation can increase the micronutrients’ availability for consumption through physiological functions, metabolism, and storage [57]. The bioavailability of micronutrients can be improved by manipulating genes involved in the uptake and translocation of Fe and Zn vis-à-vis inhibiting anti-nutrients such as phytic acid (PA) which reduces micronutrient absorption. Zn bioavailability in India is estimated to be 23% for all ages and 30% for pregnant and lactating women. As per the daily recommended dietary allowance (RDA), an adult male requires 17 mg/day Zn and 19 mg/day Fe, whereas a female requires 13.2 mg/day Zn and 29mg/day Fe. [58,59]. According to the National Sample Survey Organization, a cereal-based diet provides 70% of the Fe requirement, while other sources provide only 1% [59].

Various genes have been reported to be associated with the uptake, translocation, and transfer of Zn and Fe from soil to seed. Furthermore, micronutrient uptake is highly influenced by genotype × environment (G×E) interaction. Biofortification traits are generally polygenic in nature and there are many genes/QTLs, along with interaction with the environment, that affect the biofortification character. All the characters associated during the process of biofortificationsuch as absorption from the soil, movement in plant parts, and re-mobilization to grain are polygenic and controlled by many genes. Apart from genotypic variation for grain, Fe concentration in the soil and environment also statistically affects these traits. [8]. The secondary gene pool of wheat, e.g., *Triticum monococcum*, *Triticum boeoticum*, *T. turgidum dicoccoides*, *Aegilops tauschii, T. spelta*, and *Triticum polonicum*, has shown an ample magnitude of genetic variability for Fe and Zn content and the utilization of *T. turgidum* ssp. *dicoccoides* for improving the grain’s Fe and Zn content is well documented [8]. Zn bioavailability from a biofortified wheat diet (~95% extraction rate) was 72% higher than the unfortified wheat diet in adult Mexican women [60]. Certain anti-nutritional factors such as PA, oxalates, and polyphenols chelate Fe and Zn to form insoluble complexes reducing their bio-availabilities [60,61]. The genetic origin of wheat varieties influences the mineral bioavailability of Zn, Mg, and Cu in rats [62]. The phosphates in PA that are negatively charged form complexes with metallic cations viz., potassium (K), Mg, Fe, calcium (Ca), and Zn to form phytin, which leads to decreased bioavailability of micronutrients in humans [63]. Increasing the promoters and reducing the inhibitors will augment micronutrient bioavailability [64]. Hambidge et al. [65] studied the influence of dietary phytate on the amount of zinc absorbed in pregnant and lactating women having a higher phytate consumption of 2200 mg phytate/day. The development of varieties having a higher phytate degrading potential may cause rapid phytate degradation in the human stomach [63]. Despite significant amounts of phytate in the diet, zinc assimilation increased during late pregnancy and early lactation [65]. Finkelstein et al. [66] investigated the effectiveness of iron-rich pearl millet in increasing hemoglobin, serum ferritin (SF), and total body iron (TBI) in 246 children aged 12 to 16 in Maharashtra, India. It was discovered that by eating biofortified pearl millet for iron, the status of iron improved fourfold in children. Therefore, the bioavailability of Zn and Fe in targeted populations’ diets can be improved using biofortified wheat.

## 4. Genetics and Breeding for Biofortified Wheat

Currently, breeding strategies primarily focus on transferring genes for Fe and Znregulation from *Triticum dicoccon* and *Triticum spelta*-derived synthetics, landraces, and other high Zn and Fe germplasm into elite wheat genotypes [26]. This traditional breeding strategy incorporates several novel grain Zn alleles in elite, high-yielding germplasm. Two major approaches have been used for understanding the genetics of micronutrients (including Zn and Fe) in cereals: (i) linkage-based interval mapping (IM), and (ii) LD-based genome-wide association study (GWAS). These studies’ findings identified several candidate genes for micronutrients.

### 4.1. Quantitative Trait Loci (QTLs) for Zn and Fe

A number of researchers have developed and utilized many bi-parental mapping populations for interval mapping. Table 2 summarizes the findings of these geneticsalong with reported QTLs for Zn and Fe. Rathan et al. [67] phenotyped 189 recombinant inbred lines (RILs) from crosses involving Kachu and Zn-Shakti as the parents to determine grain Fe and Zn content and identified the genomic regions for biofortification and agronomic traits using Diversity Arrays Technology (DArT). Previous studies have identified pleiotropic QTLs for Fe and Zn content on chromosome numbers 2B, 3B, 3D, 4B, and 5A [67,68,69,70,71]. QTLs associated with Zn content were also identified in RILs from PBW343/Kenya Swara [69]. Two new QTLs for Zn were identified on the centromeric region of 2B and long arm of 3A, and 2Bc QTL from PBW343also exhibits a pleiotropic effect for 1000 seed weight. Crespo-Herrera et al. [70] concluded that a stable QTL was present at 4BS for GZn and GFe, and was linked with 1000 seed weight underlining that developing lines with high GZn and GFeare realistic without compromising grain boldness. Velu et al. [72] confirmed that two significant QTLs on chromosomes 2 and 7 are the genes that control nutrient uptake, mineral translocation, and mineral sequestration in wheat. As a result, the discovery of new pleiotropic regions for Zn and Fe concentration has increased the availability of QTLs that could be used for the concurrent improvement of Fe and Zn in crops. The QTL QZnC-1B.1 in the region where genes encoding serine–threonine/tyrosine-protein kinase are located is responsible for the Zn channel and transporter activation.

Wang et al. [73] formedtheir study in nine environments usinga high-density Affymetrix 50K SNP array to locate QTLs for grain Zn and grain Fe content in 254 RILs’ population of cross (Jingdong 8/Bainong AK58). Seven GZn QTLs that were located on seven different chromosomes (1DS, 2AS, 3BS, 4DS, 6AS, 6DL, and 7BL) explained 2.2–25.1% of variation, while four QTLs for Fe content were located on four chromosomes (s 3BL, 4DS, 6AS, and 7BL) with 2.3–30.4% of variation. The QTLs that were present on 4DS, 6AS, and 7BL showed the pleiotropic effects for Fe and Zn content in the studied germplasm. Associated SNP markers were further converted to KASP markers for breeding selection improvement of Zn and Fe.

Krishnappa et al. [74], from their study on 163 RILs, developed from WH542 a synthetic derivative that reported the genetic regions with high GFeC, GZnC, grain protein content (GPC), and thousand kernel weight (TKW). GPC had the maximum number of QTLs (10 QTLs), followed by GZnC (6 QTLs), GFeC (3 QTLs), and TKW (1 QTL) (2 QTLs). In two or more environments, four new and steady QTLs (QGFe.iari-7D.1, QGFe.iari-7D.2, QGPC.iari-7D.2, and QTkw.iari-7D) were reported. Two new pleiotropic genomic regions in chromosome 7D, flanked between Xgwm350-AX-94958668 and Xwmc550-Xgwm350, were discovered to contain co-localized QTL affecting two or more characters. A total of 11 RILs with favorable QTL combinations identified (8 for GZnC and 3 for GPC) can be used in the breeding program to develop genotypes with high Fe and Zn content along with proteins.

### 4.2. Marker Trait Associations (MTA)

Many genetic studies involving QTL mapping have been carried out to study the genetic basis of grain Zn and Fe concentrations [71]. On the other hand, the conventional mapping approach (QTL mapping approach) is restricted to the bi-parental population utilized in the study and finds the positions with low resolutions. Wheat GWAS have been utilized to understand the genetic mechanisms of quantitative traits [76]. The GWAS provides identification of high resolution QTLs with better allele coverage and the approach is also not limited to the type of population. For investigation of quality traits, few studies using GWAS have been conducted with regards to wheat [77]. According to Arora et al. [78], lineage 2 (ssp. strangulata) had better Fe and Cu concentrations compared to lineage 1 (subspecies tauschii). The number of genotyping-by-sequencing (GBS) markers applied on 114 unique *Ae. tauschii* accessions was 5249, and the GWAS was performed which helped in the identification of five associations for Fe and three associations for Zn concentrations in grain across all seven chromosomes. These associations were related to genes that encode for transcription factor regulators, transporters, and PS synthesis.

The Fe MTA AT45556 gene was discovered on chromosome 1D near the ADP-ribosylation factor (ARF) gene, which is related to the vital movement of molecules in cells and is associated with daily changes in the oozing of mugineic acid family phytosiderophores (MAs) [79,80,81]. A candidate gene for Fe content is located on chromosome 7D with marker AT2276 transcripts and an AT-hook motif nuclear-localized protein that regulates gene expression. On chromosome 4D, the Zn MTA AT65984 located next to HVA22, which is an abscisic acid-induced protein, hinders gibberellin (GA)-mediated programmed cell death in crops’ aleurone cells and performs as an enhancer for accumulation of metals under unfavorable conditions [81]. AT2707 on 2D is associated with Zn content and is located near the predicted Scarecrow-like 3 (SCL3) GRAS transcription regulator, which acts as an enhancer for the integrated efficient GA pathway [82]. AT2707 is also an associated ABC transporter which is involved in exporting or importing a range of substrates, from micro to macromolecules. Furthermore, a Zn MTA (AT77346 located on Chromosome 6D) was related to a Malonyl-coenzyme A: anthocyanin 3-O-glucoside-60 0-Omalonyl transferase gene.

Cu et al. [83] investigated the genetics of micronutrients in the grain and rachis during the development of grain and physiological ripeness using a Harvest Plus Association Mapping (AM) panel. In developed grain, 72 MTAs were notably related to Zn concentration and 65 MTAs to Fe concentration. For mature grain, significant pleiotropic effects were observed on 1A, 3B, and 5B, with the marker located on 5B at 95.5 cMand remaining stable over two crop years of testing. The most significant MTAs for Fe concentration were found on chromosomes 5A and 5Bduring the years 2014–15 and chromosome 7B in 2015–16 (LOD score more than 4.9). The study also helped in co-localized MTA for Fe and Zn concentrations in various grain-filling stages on multiple chromosomes. Putative candidate genes controlling metal uptake, transportation, and storage protein processing were reported in the identified genomic regions. Wang et al. [73] identified five MTAs’ loci for Fe that accounted for 10% of the variance on chromosomes 6B and 7B and three for Zn that accounted for >10% of the variance on 3B and 4B. Liu et al. [84] applied GWAS in 161 breeding lines of wild emmer wheat to describe grain iron, zinc, and manganese concentrations (GFeC, GZnC, GMnC). Using both the general linear and the mixed linear affects, the results revealed 14 important MTAs linked with GFeC, GZnC, and GMnC. Six MTAs were located on 3B, 4A, 4B, 5A, and 7B and were appreciably associated with GFeC. Table 3 shows that three MTAs on 1A and 2A were linked with zinc concentration.

### 4.3. Meta-Quantitative Trait Loci (MQTLs)

Meta-QTL analysis (MQTL analysis) is a reliable approach for combining available data on QTLs from different mapping populations that helps to understand the genetic mechanism of quantitative characters. Shariatipour et al. [89] used a meta-analysis in wheat with seven independent segregating populations to find the most durable QTLs for traits such as yield, quality characters, and micronutrient concentrations. They observed that QTLs coding for Zn and concentration were located together (57.1%), implying the possibility of simultaneous improvement of both the nutritional traits. During the last thirteen years, altogether 735 QTLs from 27 independent mapping populations were used for the meta-QTL analysis. The findings revealed that 449 QTLs were successfully predicted onto the genetic consensus map, resulting in 100 meta-QTLs that were distributed among wheat chromosomes. MQTL 3B 1, which consisted of 43 loci, had the maximum number of QTLs among the identified MQTLs; another meta QTL 7A 3 contained 29 QTLs. The meta-QTLs identified in this investigation will assist in locating CGs in these areas that are liable to traits of economic importance and generating allele-specific markers for marker-assisted selection applications [90,91]. In wheat and its relatives of different ploidy levels, such as *T. monococcum* (2×) and *T. boeoticum* (2×), *T. dicoccoides* (4×) and *T. durum* (4×), or hexaploid wheat *T. aestivum* (6×) sources, approximately 25 QTLs on 16 different chromosomes were found. Genetic regions having QTLs on chromosomes 1B, 2B, 3A, 4B, 5B, 6B, and 7A appear promising (Table 4).

## 5. Scaling up Biofortified Wheat Varieties in India

Targeted improvement for nutritional traits has led to the release or testing of 393 nutria-rich crop varieties in 63 nations across the globe and has been proved to be beneficial for 48 million populace [92]. Realizing the prominence of the nutritional quality of wheat, research efforts were streamlined to develop and release a series of biofortified varieties. These biofortified varieties were wellequipped to provide the required micronutrients for adequate growth and development. During 2021, 14 biofortified wheat varieties (less than five years old) were included in seed chains, and the highest breeder seed production was carried out in DBW187 (2315q) followed by HD8759 (420q) and DDW 47 (269.10q). During 2020–21, 14 biofortified wheat varieties contributed 19.54% (3334.60q of 17066.35q) of the total breeder seed indent, whereas 3890.60q of breeder seed of 14 biofortified varieties was produced for further multiplication (Table 5) [93].

**Figure 3:**
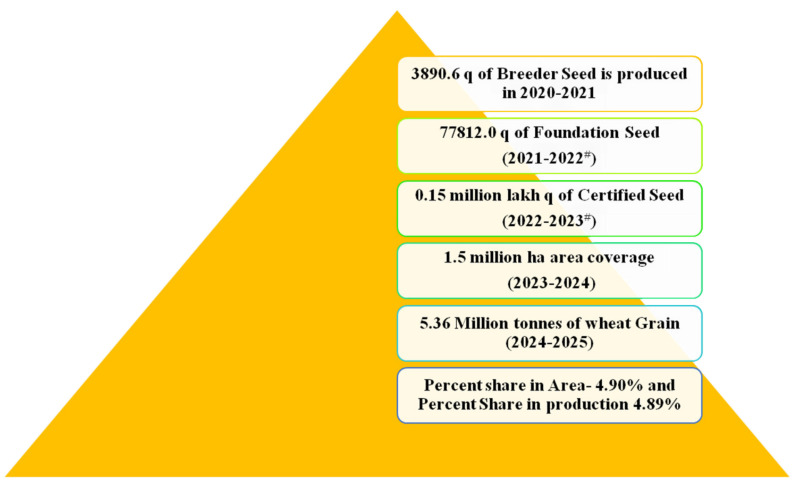
Projected impact of biofortified wheat @ 100% conversion of breeder seed into certified seed (# percent share in area and production is calculated as per the fourth advance estimate during 2020–2021 of wheat area (31.61 M ha); production (109.52 million tons), productivity of 3464 kg/ha. andseed rate@100 kg/ha).

Assuming 100% conversion of breeder seed of these varieties into foundation and certified seed in subsequent years will result in production levels of 0.15 million quintals of certified seed in 2022–2023. This certified seed will cover an area of 1.5 million ha in 2023–2024, with 5.32 million tons of wheat grains available for consumption during 2024–2025 (Figure 3).

Given the potential for biofortified varieties to address specific nutrient deficiencies without significantly changing dietary habits and cropping patterns, there is scope for promoting these varieties among farmers. It is necessary to formulate an apt strategy for promoting biofortified varieties. The study of these varieties must account for diverse aspects, such as identifying suitable biofortified varieties to address micronutrient deficiencies, creating awareness among consumers and farmers, technological backstopping, and planning policy support [94]. Talsma et al. [95] concluded that consumers would pay higher prices for certain biofortified crops considering health benefits. Increased production alone of biofortified varieties will not be sufficient; integration of agriculture and nutrition programs provide a greater opportunity for combating malnutrition [96]. Including biofortified varieties in nutrition schemes and premium prices will help maintain biofortified varieties. Establishing biofortified wheat varieties under the National Food Security Mission and further linking with the Integrated Child Development Scheme (ICDS) and other nutrition schemes of the health department will ensure a better reach of the targeted population.

## 6. Conclusions

Wheat contributes nearly one fifth of total daily dietary energy requirement globally and biofortified varieties can be a proven source for improving nutritional health. Several studies have found that there is enough genetic variability available in wheat to support the program on the breeding of micronutrients. As a result, targeted breeding for increased Zn and Fe could significantly impact the development and release of biofortified wheat varieties in India. The availability of high Zn nurseries and locally bred materials evaluated at multiple environments to identify biofortified varieties with high Zn, Fe, and protein concentrations has resulted in the release of several nutri-rich varieties in recent years. Understanding the genetics and physiology of grain Zn and Fe is imperative to fully exploit the benefits of biofortification; thus, this review examined the physiological aspects of wheat biofortification and the genetic control of these traits in different mapping and genetic studies. Further, it underlines the importance of a robust seed system for rapidly disseminating biofortified varieties, benefitting farmers and end-users.

## Figures and Tables

**Table 1:** Baseline level achieved by biofortification in wheat (Indian scenario).

Nutrients	Baseline Level	Level Achieved by Biofortification	Target
Protein	8–10 (%)	More than 12.0 (%)	12–15 (%)
Iron	28–32 ppm	More than 38 ppm	40–50 ppm
Zinc	30–32 ppm	More than 40 ppm	40–50 ppm

**Table 2:** Identified QTLs for grain Zn and concentration in different mapping populations in bread wheat (*Triticum aestivim*. L).

S. No.	Pedigree	No. of QTLs	Ref.
	Cross Type (Number)	Zn Fe	
1.	Kachu/Zinc-Shakti RILs (190)	09 04	[67]
2.	Jingdong 8//Bainong AK58 RILs (254)	07 04	[73]
3.	WH542/synthetic derivative (*Triticum dicoccon* PI94624/*Aegilops tauschii* RILs 163) (409)//BCN)	06 03	[74]
4	No. of identified QTLs since 2009–2019 in different mapping populations	111 93	[75]
	Total no. of identified QTLs	133 104	

**Table 3:** Identified MTAs in *Triticum aestivum* L. and *Ae. tauschii* using GWAS.

S. N.	Association Panel (Size)	Location(s)	Environments	Method for Estimation	Markers	MTAs	Ref.
						Zn Fe	
1	HPAM Panel (330)	India, Mexico	6	EDXRF	14,273	39 -	[72]
2	Chinese winter wheat grain (205)	China	4	ICP-MS	24,355	3 5	[73]
3	*Ae. Tauschii* panel (114)	India	3	ICP-OES	5249	4 5	[78]
4	HPAM Panel (330)	Mexico	2	ICP-MS	28,074	72 65	[83]
5	HPAM panel (330)	Mexico	3	ICP-MS 7500x	28,074	5 --	[83]
6	CN16 x D1-Wild emmer wheat-advanced lines (161)	China	4	PinAAcle 900T, USA	13,116	3 6	[84]
7	EuWV Panel (369) Sub-panel (183)	Germany	3 3	ICP-OES ICP-OES	15,523 28,710 (Zn) 44,233 (Fe)	40 41 161 - - 137	[85]
8	SHW (123)	Turkey	2	ICP-MS	35,648	13 03	[86]
9	SWRS (246)	India	2	EDXRF	-	94 33	[87]
10	SHW (Longdon × 47 *Ae tauschii*) (47)	Japan	2	ICP-AES	70 (SSRs)	03 03	[88]

**Table 4:** QTLs identified on different chromosomes in *T. aestivium* and its related species.

Chromosome No.	A-Genome	B-Genome	D-Genome
1	*T.monococcum* *T.aestivum*	*T.aestivum, T. durum*	*T. aestivum*
2	*T. durum*, *T. dicoccoides*	*T.aestivum*	*-*
3	*T.aestivum*	-	*T. aestivum*
4	*T. aestivum*	*T. aestivum*	*T. aestivum*
5	*T. aestivum*, *T. dicoccoides*, *T. monococcum*	*T.aestivum*	*-*
6	*T. spelta*, *T. aestivum*	*T. aestivum* *T. dicoccoides*, *T. durum*	*-*
7	*T. aestivum*, *T. dicoccoides*, *T. boeoticum*, *T. monococcum*	*T. dicoccoides*	*-*

**Table 5:** Breeder seed indents and production of biofortified wheat varieties during 2020–2021 in India.

S.No.	Variety	Growing Conditions	Quality Trait	Year of Notification	Breeder Seed (Quintals)	
					Indent	Production
1	DBW 187	NWPZ (IR-ES and TS) and NEPZ (IR-TS)	Fe (41.3ppm) and Zn (43.7 ppm)	2020	1617.35	2315.00
2	DDW 47 (d)	CZ (RI-TS)	Fe (40.1 ppm)	2020	155.00	269.10
3	PBW 771	NWPZ (IR-LS)	Zn (41.4 ppm)	2020	71.80	80.00
4	HD 3249	NEPZ (IR-TS)	Fe (42.5 ppm)	2020	35.80	40.00
5	PBW 752	NWPZ (IR-LS)	Protein (12.4%)	2019	71.80	77.00
6	PBW-757	NWPZ (IR-VLS)	Zn (42.3 pm)	2019	33.00	33.00
7	HI-8777 (d)	CZ (RF-TS)	Fe (48.7 ppm) and Zn (43.6 ppm)	2018	2.00	110.50
8	DBW 173	NWPZ (IR-LS)	Protein (12.5%) and Fe (40.7 ppm)	2018	170.00	198.00
9	MACS 4028	PZ (IR-TS)	Protein (14.2%), Fe (46.1 ppm) and Zn (40.3 ppm)	2018	2.00	5.00
10	UAS-375	PZ (RI-TS)	Protein (13.8%)	2018	2.00	3.00
11	PBW 1 ZN	NWPZ (IR-TS)	Fe (40 ppm) and Zn (40.6 ppm)	2017	191.00	240.00
12	WB-2	NWPZ and Bihar (IR-TS)	Zn (42 ppm) and Fe (40 ppm)	2017	80.20	90.00
13	HD-3171	NEPZ (RI-TS)	Fe (47.1 ppm)	2017	56.45	10.00
14	HI 8759	CZ(IR-TS)	Fe (41.1 ppm) and Zn (42.8 ppm)	2017	846.20	420.00
		Total			3334.60	3890.60

(NWPZ: North Western Plains Zone, NEPZ: North Eastern Plains Zone, CZ: Central Zone, PZ: Peninsular Zone, IR: Irrigated, RI: Restricted Irrigation, RF: Rainfed, ES: Early Sown, TS: Timely Sown, LS: Late Sown, VLS: Very Late Sown).

## Data Availability

Not applicable.
